# Medium-sized tandem repeats represent an abundant component of the *Drosophila virilis* genome

**DOI:** 10.1186/1471-2164-14-771

**Published:** 2013-11-09

**Authors:** Murat A Abdurashitov, Danila A Gonchar, Valery A Chernukhin, Victor N Tomilov, Julia E Tomilova, Natalia G Schostak, Olga G Zatsepina, Elena S Zelentsova, Michael B Evgen’ev, Sergey Kh Degtyarev

**Affiliations:** 1 SibEnzyme Ltd., Ak. Timakova Str. 2/12, Novosibirsk 630117, Russia; 2 Engelhardt Institute of Molecular Biology, Vavilov str. 32, Moscow 119991, Russia; 3 Institute of Cell Biophysics, Pushchino 142290, Russia

**Keywords:** *Drosophila virilis*, *in silico* digestion, Medium size repeats, Evolution

## Abstract

**Background:**

Previously, we developed a simple method for carrying out a restriction enzyme analysis of eukaryotic DNA *in silico*, based on the known DNA sequences of the genomes. This method allows the user to calculate lengths of all DNA fragments that are formed after a whole genome is digested at the theoretical recognition sites of a given restriction enzyme. A comparison of the observed peaks in distribution diagrams with the results from DNA cleavage using several restriction enzymes performed *in vitro* have shown good correspondence between the theoretical and experimental data in several cases. Here, we applied this approach to the annotated genome of *Drosophila virilis* which is extremely rich in various repeats.

**Results:**

Here we explored the combined approach to perform the restriction analysis of *D. virilis* DNA. This approach enabled to reveal three abundant medium-sized tandem repeats within the *D. virilis* genome. While the 225 bp repeats were revealed previously in intergenic non-transcribed spacers between ribosomal genes of *D. virilis*, two other families comprised of 154 bp and 172 bp repeats were not described. Tandem Repeats Finder search demonstrated that 154 bp and 172 bp units are organized in multiple clusters in the genome of *D. virilis.* Characteristically, only 154 bp repeats derived from *Helitron* transposon are transcribed.

**Conclusion:**

Using *in silico* digestion in combination with conventional restriction analysis and sequencing of repeated DNA fragments enabled us to isolate and characterize three highly abundant families of medium-sized repeats present in the *D. virilis* genome. These repeats comprise a significant portion of the genome and may have important roles in genome function and structural integrity. Therefore, we demonstrated an approach which makes possible to investigate in detail the gross arrangement and expression of medium-sized repeats basing on sequencing data even in the case of incompletely assembled and/or annotated genomes.

## Background

Though multiple plant and animal genomes have been sequenced and annotated, including many from *Drosophila*, abundant fractions of repeated DNA often forming heterochromatic regions of the genome escape description. Large heterochromatic segments of genomes remain poorly analysed because the repetitive nature of the DNA present in heterochromatin makes cloning, assembly and annotation very difficult. Heterochromatic regions are the dark matter in genomes and even for well studied organisms we still do not have a complete genomic sequence due to the difficulties of sequencing these regions. Previously, we developed a simple method for carrying out a restriction enzyme analysis of eukaryotic DNA *in silico*, based on the known DNA sequences of the genomes [1]. This method allows the user to calculate lengths of all DNA fragments that are formed after a whole genome is digested at the theoretical recognition sites of a given restriction enzyme. The program also constructs distribution diagrams of the calculated restriction DNA fragments. These distribution diagrams display distinct peaks, where DNA fragments of definite lengths are present due to DNA repeats in eukaryotic genomes. A comparison of the observed peaks in distribution diagrams with the results from rat, mouse and human DNA cleavage using several restriction enzymes performed *in vitro* have shown good correspondence between the theoretical and experimental data [2,3]. Here, we applied this approach to the annotated genome of *Drosophila virilis*.

Satellite and minisatellite DNAs constitute a considerable part of the genomic DNA and are often found as runs of thousands or more copies of unit sequences (100-300 bp and 3–15 bp, respectively) predominantly localized in heterochromatic regions. SatDNA is generally formed by long tandem arrays in which the units are repeated in a head-to-tail fashion [4,5]. More than 40% of the *D. virilis* genome consists of three simple minisatellite DNAs, each of which are seven base pairs long, that are located predominantly in pericentromeric heterochromatin in all chromosomes of species within the *virilis* phylad [6].

*D. virilis* represents the most karyotipically primitive species of the *virilis* phylad [7,8]. The availability of a sequenced genome enables the application of our *in silico* digestion method to look for the presence and abundance of repeats that were not adequately described in the sequenced genome of this species, due to limitations of current sequencing and mapping techniques for assembling tandem repeat motifs scattered throughout the genome.

In past decades, an extensive analysis of various classes of repeats, including cryptic satellites and various classes of mobile elements in *D. virilis* and other species of the *virilis* group, have been performed in our laboratory and by other groups [9-14]. Therefore, it was of significant interest to extend our analysis to the repeated fraction of the *D. virilis* genome and to explore *in silico* digestion in combination with conventional restriction analysis. These analyses help to reveal and describe uncharacterized and highly abundant families of repeats within the genome of this unique in many ways [6,7], species of *Drosophila*. Here, we describe three highly abundant families of medium-sized tandem repeats within the *D. virilis* genome. The consensus repeat units comprising these families were cloned, sequenced and compared with those of the related species *D. americana*. This analysis emphasizes the validity and versatility of the *in silico* digestion method in studying various repeats often included in the heterochromatic fraction of genome.

## Results and discussion

### Several families of medium-sized tandem repeats are revealed by *in vitro* and *in silico* restriction analysis in *D. virilis* genomic DNA

**Figure 1:**
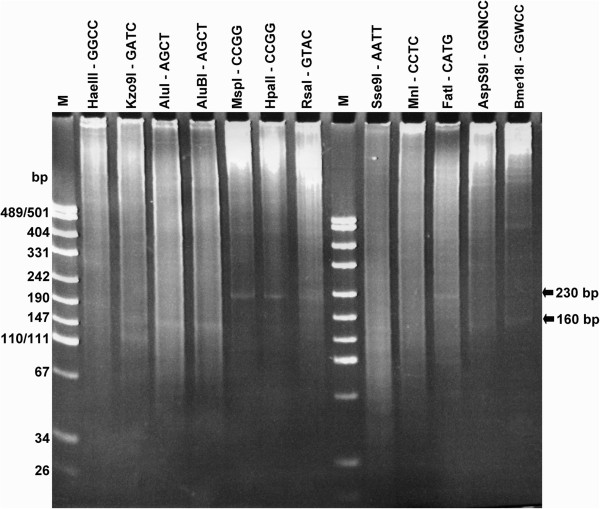
**Electrophoretic separation of *D. virilis *** **DNA fragments produced by restriction enzyme hydrolysis.**

We performed hydrolysis of *D. virilis* genomic DNA with different restriction endonucleases. Figure 1 shows the patterns of *D. virilis* DNA cleavage with 12 restriction endonucleases. According to the data presented in Figure 1, DNA hydrolysis with restriction enzymes results in the formation of a few distinct visible bands.

Interestingly, some of the restriction endonucleases produce DNA fragments of the same length. For example, a DNA fragment of ~230 bp is observed after DNA hydrolysis with *Msp*I/*Hpa*II, *Rsa*I and *Fat*I, whereas ~160 bp DNA fragment is clearly observed after digestion with *Kzo*9I, *Alu*I/*Alu*BI, *Asp*S9I and *Bme*18I (Figure 1).

The results of our study as well as genome sequencing data show that several families of abundant repetitive elements of medium size exist in the *D. virilis* genome. Moreover, the presence of same-size fragments in patterns of DNA cleavage with different restriction enzymes is a clear indication that these repetitive sequences are arranged in tandem. Therefore, based on the experimental data depicted in Figure 1, the tandem repeats of approximately 160 bp and 230 bp in length are present in the *D. virilis* genome in high copy number.

Satellite DNAs, which form heterochromatin regions in eukaryotic genomes, are the major source of tandem repeats in most of the genomes studied. However, *Drosophila* satellite DNA with a few prominent exceptions, includes very short repetitive sequences of 4–14 bp in length, depending on the species [4,9,15].

We analyzed a known structure of the *D. virilis* genome to find medium-sized tandem repeat candidates. We performed *in silico* DNA digestion of the currently available draft *D. virilis* genome sequence, using recognition sites for the restriction endonucleases *Alu*I, *Kzo*9I and *Hpa*II, according to the earlier published protocols [1]. These restriction enzymes recognition sequences were chosen because they produce clearly visible DNA fragments.

**Figure 2:**
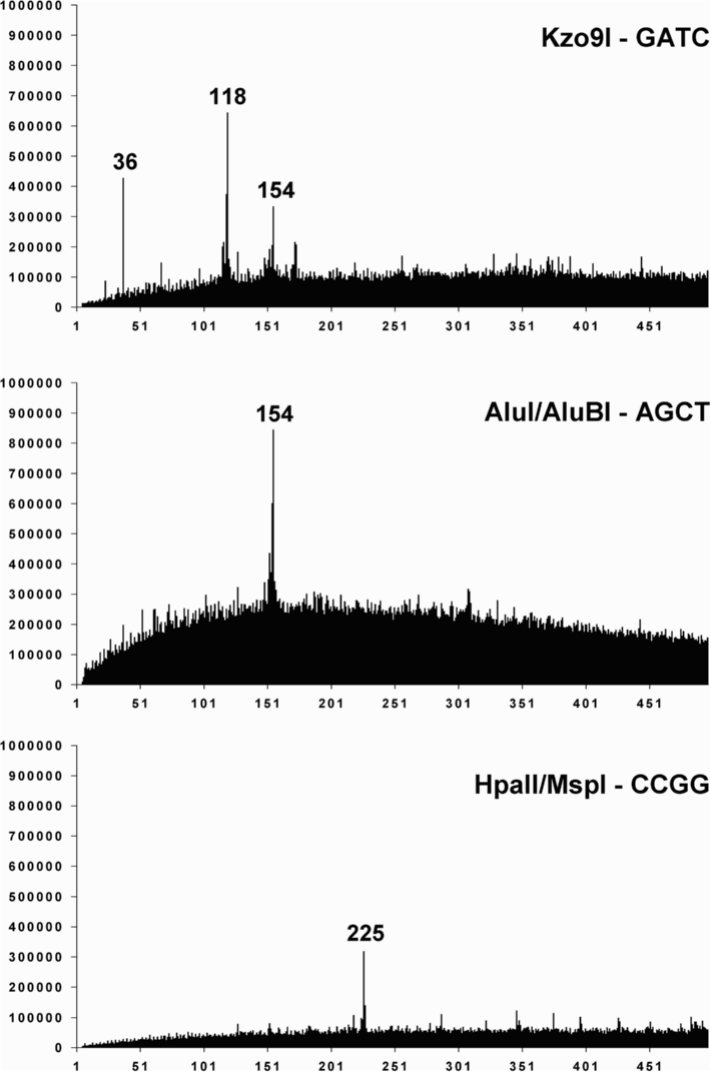
**Distribution diagrams of *D. virilis *** **total DNA fragments lengths (expressed in bp) depending on the fragment size resulting from DNA digestion *in silico.***

Figure 2 summarizes the distribution of the fragment lengths obtained in the *in silico* digestion. According to the distribution, DNA hydrolysis with *Hpa*II results in the formation of a DNA fragment of 225 bp in length, *Alu*I hydrolysis produces a 154 bp DNA fragment and *Kzo*9I digestion gives three distinct DNA fragments that are 36, 118 and 154 bp in length. These data correspond to the experimental results presented in Figure 1, except the 36 bp fragment.

It is noteworthy that DNA fragments that are shorter than 100 bp are not usually observed on the gel (Figure 1) because their combined molecular mass remains below detection level [1].

**Figure 3:**
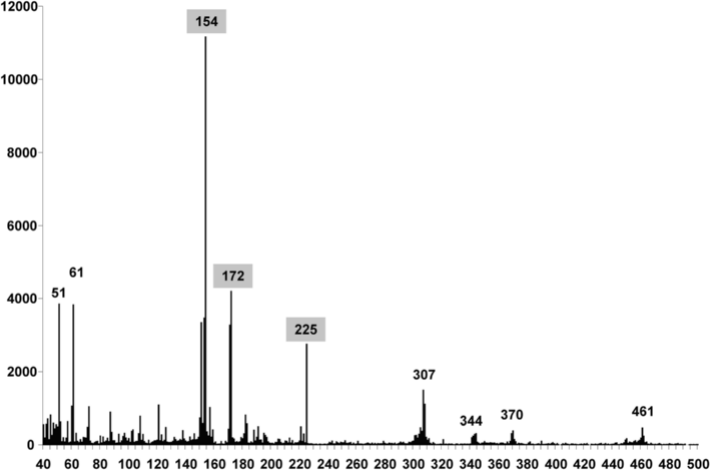
**The quantity of tandemly arranged repeats of 40–500 bp length in the *D. virilis *genome.** Abscissa axis - length of fragment in bp; ordinate axis - number of copies. The major peaks are indicated above the diagram.

Independently, we scanned the *D. virilis* genome using the Tandem Repeat Finder software to find tandemly arranged repetitive elements that were 40–500 bp in length (Figure 3).

A comparison of the diagrams in Figures 1, 2 and 3 shows consistent results achieved by the three independent approaches, except the presence of an additional DNA peak that is 172 bp in length and clearly observed in Figure 3.

Therefore, according to our results, there are multiple tandem repeats in the *D. virilis* genome that are much longer than the previously described minisatellite sequences, and they are unrelated to the pvB370 satellite family and pDv family described in this species [9,15]. The origin and genomic location of 154, 172 and 225 bp fragments that comprise significant parts of the *D. virilis* genome are discussed below.

### 225 bp tandem repeats represent intergenic spacers between ribosomal genes

**Figure 4:**
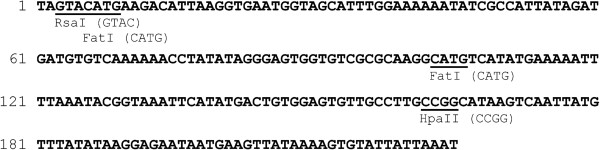
Recognition sites of three restriction enzymes in the consensus sequence of the 225 bp tandem repeat.

The ribosomal DNA (rDNA) of insects contains several hundred structural-functional units arranged in tandemly repeated clusters in nucleolus organisers, separated by several transcribed and nontranscribed spacers. Tandem repeats of 225 bp in DNA of *D. virilis* have been noted elsewhere [16]. These 225 bp repeats are located in IGS (intergenic spacer) between 28S and 18S rRNA genes. It was suggested that, in *Drosophila*, these repeats are not transcribed and most likely serve as enhancers of gene expression [17]. Ribosomal RNA genes in *Drosophila* form clusters that are abundant (i.e., several hundreds of copies) within the genome. Each *Drosophila* species contains tandem repeats of defined length within an IGS region [18]. Figure 4 shows a consensus DNA sequence of a *D. virilis* IGS tandem repeat (225 bp), with highlighted recognition sites of restriction endonucleases *Rsa*I, *Fat*I and *Hpa*II/*Msp*I.

There are unique sites for *Rsa*I and *Hpa*II/*Msp*I within the IGS tandem repeat, and its cleavage with the indicated restriction enzymes should result in the formation of 225 bp DNA fragments and thus correspond to the experimentally observed data (Figure 1). Surprisingly, the consensus IGS tandem repeat contains two sites recognized by the *Fat*I restriction enzyme.

**Figure 5:**
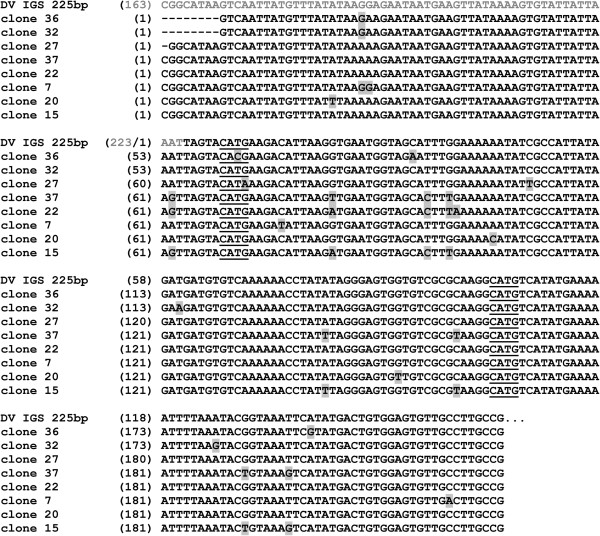
**Alignment of cloned 225 bp *HpaII *fragments and the consensus sequence of the 225 bp tandem repeat from *D. virilis *rDNA IGS.** Nucleotides that differ from the corresponding ones in the consensus sequence are marked in blue. The part of the consensus sequence (163–225 bp, green colour) was transferred to the beginning for comparison. *FatI* sites are underlined.

To confirm the origin of the visible fragments, we have purified the 225 bp *Hpa*II fragments from the gel for cloning and sequencing. Eight of the twenty-eight obtained sequences exhibit a high degree of similarity (96-99% identity) to the 225 bp consensus sequence (Figure 5) while the remaining twenty sequences exhibit no significant homology to the consensus (data not shown).

According to Figure 5, two of the eight sequenced *Hpa*II fragments carry a mutation in the first *Fat*I recognition site, which may explain the presence of a 225 bp DNA fragment in the hydrolysis products from this enzyme.

**Figure 6:**
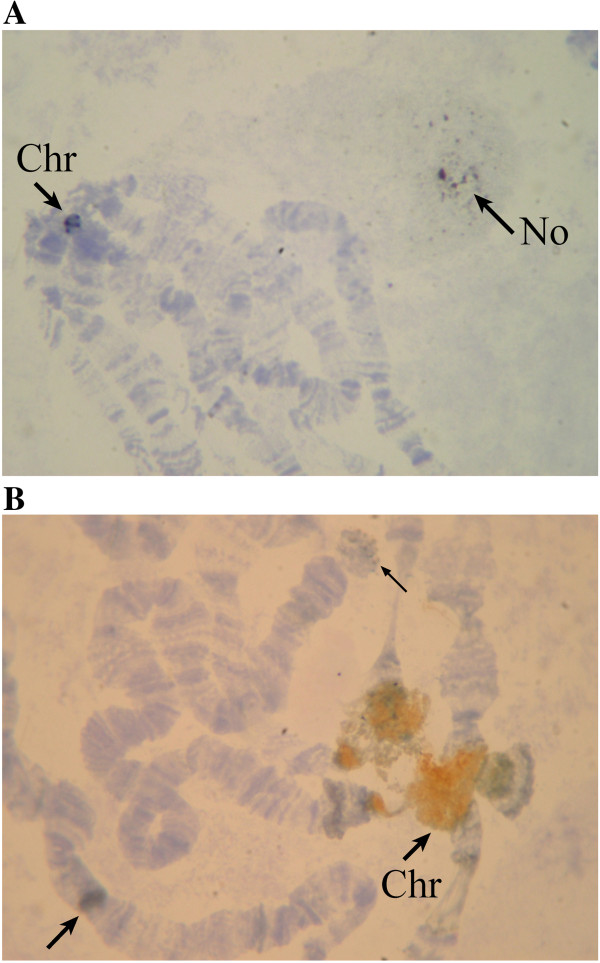
***In situ *hybridisation of *D. virilis *polytene chromosomes with the tandem repeats. (A)** 225 bp fragment. Arrows indicate grains in the chromocenter (**Chr**) and scattered grains in the nucleolus (**No**). **(B)***Helitron* fragment (350 bp). Arrows indicate strong labelling in the chromocenter (**Chr**) and multiple sites of hybridization in the chromosomes.

We performed an *in situ* hybridization of a 225 bp *Hpa*II probe (plasmid p*Hpa*V-kl22) with *D. virilis* salivary gland polytene chromosomes; as expected, we observed significant hybridization in the heterochromatic chromocenter and multiple diffuse grains in a restricted region of the nucleolus (Figure 6A). Our Northern hybridization experiments using total RNA and labelled 225 probe demonstrated that these repeats are most likely not transcribed in *D. virilis* because we did not observe any transcription in the *D. virilis* strain used for analysis (strain 160) of the tandem repeat and only weak transcription in another *D. virilis* strain (strain 9), which probably represents a read-through transcription of this repeat. Furthermore, we failed to observe any hybridization with poly (A)-RNA of both *D. virilis* strains in Northern blots using the labeled 225 probe (data not shown).

### The 154 bp tandem repeat family is apparently derived from *Helitron* transposable element

**Figure 7:**
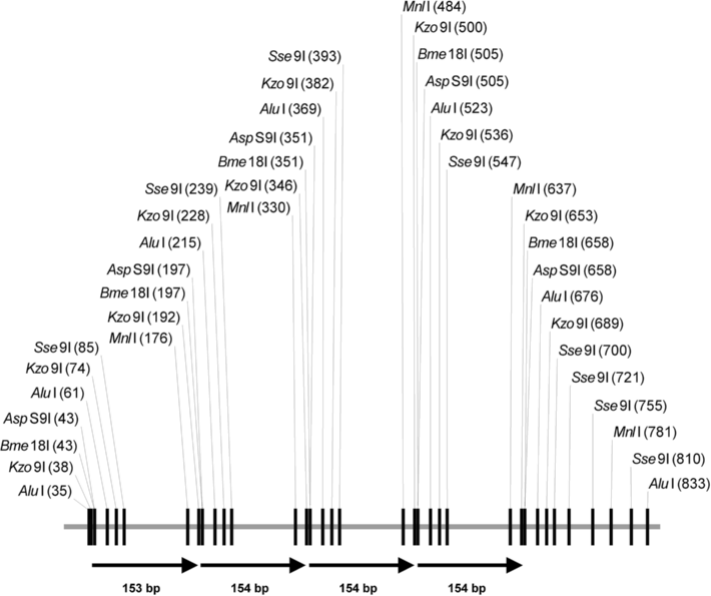
**Map of the *Helitron*-2 fragment that contains tandem repeats.** Tandem repeat units are indicated by arrows. The sizes of the units comprising the cluster are given.

Tandem repeats of 154 bp, as indicated in Figures 1, 2 and 3, are not described in the literature to our knowledge. However, results from the Tandem Repeat Finder show that there are 2219 clusters in the genome that contain 153–154 bp tandem repeats. There are approximately 14160 individual repeat units of this length. To determine the origin of the 153–154 bp repeat, we have extracted all consensus sequences from the table produced by the Tandem Repeat Finder and assemble them in one consensus sequence. It is of note, that 118 bp and 36 bp fragments in *Kzo*9I distribution diagram apparently appear due to hydrolysis of 154 bp fragment and further analysis (see Figure 7) confirms this assumption.

The comparison of the consensus sequence with the REPBASE database [19,20] shows that the 153–154 bp fragment is derived from the *Helitron*-2 interspersed repetitive element. The full length of the intact *Helitron*-2 transposon of *D. virilis* is 9141 bp, and the 153–154 bp consensus sequence exhibits a high degree of homology to the region found between positions 237 and 1087. This particular region contains four copies of the 153–154 bp repeat within the full length of the consensus sequence of *Helitron*-2. A map of this *Helitron-2* fragment is depicted in Figure 7.

This consensus sequence contains two GATC sites (i.e., *Kzo*9I recognition sites) in each unit, but we still can see the presence of intact 154 bp fragments in Figure 2, which means that many 154 bp fragments include only one *Kzo*9I recognition site.

Full-length *Helitron*-2 elements are not abundant in the *D. virilis* genome, but there are a lot of truncated copies that mainly include the first 928 bp fragment 5’ of the transposon. In general, *Helitron*-2 fragments of different length occupy as much as ~5% of *D. virilis* genome [21]. Thus, the 153–154 bp DNA fragments that are visible in the gel (Figure 1) may be explained by the presence of multiple, predominantly truncated, copies of this transposon representing the remnants of the *Helitron* amplification process that occurred at some point in the *virilis* group evolution.

It is noteworthy that abundant *DINE-I* transposable element has been described in 12 species of *Drosophila*, including *D. virilis.* It was proposed that *DINE-1* is also related to *Helitrons*, a family of DNA-mediated transposons [22]. However, our analysis demonstrates that the 154 bp tandem repeats are definitely not included in *DINE-I* transposon sequences in *D. virilis*.

**Figure 8:**
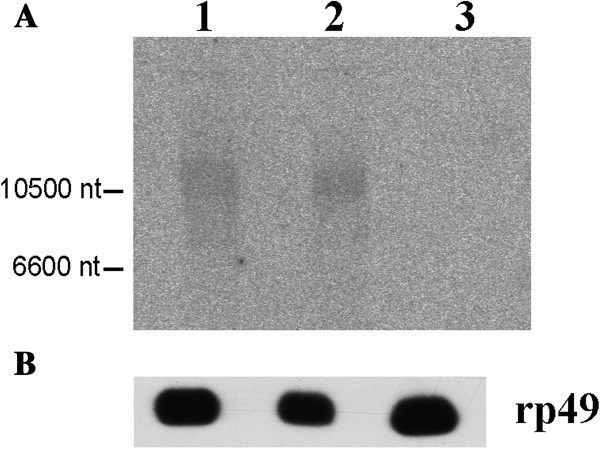
**Northern hybridization with with Poly (A) RNA isolated from *D. virilis *and *D. melanogaster *ovaries. (A)** Labeled 350 bp fragment of *Helitron*-2 was used as a probe. Lane 1: Strain 9; lane 2: Strain 160; lane 3: *D. melanogaster* strain *Df(1)*^*w67c23y*^. **(B)** The filter was stripped of the labeled probe and rehybridized with labeled *rp49*, an abundantly expressed Drosophila gene [23].

To describe the distribution of 154 bp family of repeats in the chromosomes of *D. virilis* we carried out *in situ* hybridization of salivary gland polytene chromosomes with a 350 bp probe that was obtained by PCR from *D. virilis* DNA and included two 154 bp repeat units. As expected, the experiments revealed a very strong hybridization in the chromocenter and multiple sites of hybridization scattered in the chromosomes (Figure 6B). A Northern blot analysis demonstrated that the 154 bp repeats are present in the poly (A) (+) fraction of RNA because the correspondent probe hybridized with a high molecular weight (10 kb) band in both of the *D. virilis* strains studied, but not in *D. melanogaster* (Figure 8). Although the size of the hybridization fragment corresponds to the full-size transcript of *Helitron*-2 (approximately 9 kb), it will be necessary to use other probes complementary to this transposable element to prove that *Helitron* is really transcribed in *D. virilis* species.

### Multiple 172 bp tandem repeats are located in the *ap* gene of *D. virilis* and most likely in many other sites of the genome

Surprisingly, we do not observe the 172 bp fragment in the experimental digestion (Figure 1), and by *in silico* restriction analysis (Figure 2) although an investigation of the sequenced *D. virilis* genome using the Tandem Repeat Finder revealed a high peak at this fragment length (Figure 3). It is noteworthy that there is similarity in the monomer length of many centromeric satellites (often approximately 170 bp), which leads to the assumption that such a repeat unit might reflect uniformity in nucleosome phasing and heterochromatin propagation [5]. However, we failed to find any family consisting of sequences of this length in any studied Drosophila genomes with the exception of *D.* ananassae [24].

According to our analysis the number of 171–172 bp repeats in the sequenced *D. virilis* genome is 7455 and the number of genomic clusters that contain such units is 778. We aligned most of the isolated 171–172 bp sequences and obtained the following consensus sequence:

TACCATSAAATATCCTACATAGACATAGGTCGAAAATTCCCAACCCCATAACTCGGCCAAAACTCAACCGATTTTCATAAGGTWTAMMTTTTTGTTCATGGTTTGACCTCWATATCAATCTGGCATATAAATCTGACAACTTTATTTTTGGTCAAAATTCATGTGAAAATGG.

The BLAST search for homologous sequences was performed and revealed one region of the *D. virilis* genome which contains multiple copies similar to the consensus sequence.

**Figure 9:**
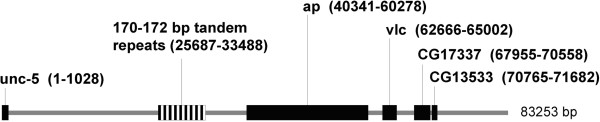
**The structure of the *apterous *** **locus in *D. virilis*** **and the location of the cluster of 172 bp tandem repeats, as shown in GenBank sequence AY186999.**

The region is located before the *apterous* (*ap*) gene of *D. virilis* (GenBank acc.no. AY186999). In *D. melanogaster*, this gene contains a homeodomain and encodes a key developmental regulatory protein [25]. In *D. virilis*, this genetic region contains 29 tandem units 172 bp in length, as well as other homologous sequences of different lengths. The general organization of this region in the latter species is depicted in Figure 9.

The structure and sequences of individual repeats included in the *apterous* cluster and their alignment are summarised in Additional file 1: Figure S1. The analysis indicates that the cluster of 172 bp tandems located near the *apterous* gene has a rather complex structure. Blocks of 172 bp repeats (2–6 units) are interrupted by sequences of 161 bp and 28–29 bp in length, which represent the fragments of the same basic 172 bp consensus sequence. Short 28–29 bp fragments always end with a hexanucleotide motif that is not homologous to the consensus sequence; after this motif, the hexanucleotide 161 bp fragment lacking 11 bp at the 5’ end is always observed. All 172 bp units contained in the cluster exhibited amazingly high levels of identity (Additional file 1: Figure S1), which suggests the concerted evolution of the sequences. Furthermore, the whole cluster, except for 29 full-size 172 bp units, contains 12 5’-deleted copies of the consensus sequence, 13 fragments 28–29 bp in length and single fragments that are 170, 173 and 175 bp in length.

According to our Tandem Repeat Finder analysis, there are other clusters comprised of homologous 171–172 bp tandem repeats in the *D. virilis* genome, but the absence of a well annotated genome prevents the determination of their locations. We do not yet know whether the described 172 bp cluster has something to do with *apterous* function, and we did not find any relevant information in the literature [26]. The role of this family of tandemly arranged sequences may also include regulation of gene activity, as in the case of the 225 bp tandem repeats.

Unlike the 154 bp and 225 bp tandem repeats, DNA fragments of 171–172 bp in length isolated from different genomic regions (data not shown) display a high level of variability in the sequence. This difference may explain why the band that corresponds to the 171–172 bp fragment was not present in the experimental (Figure 1) and *in silico* digestion (Figure 2).

### Investigation of the three major tandem repeats families in the genome

#### *D. americana*, another species of the *virilis* group

*D. americana* belongs to the *virilis* phylad of the *virilis* group and is separated from *D. virilis* by 4–5 million years of divergent evolution [7,8,14]. Given the evolutionary relationship between *D. virilis* and *D. americana* we were interested in comparing the abundancy of the medium-sized repeats within the genomes of these two species. The *D. virilis* which basing on most primitive karyotype lacking intraspecific rearrangements, maximal content of satellite DNA among species of the group and many other features appears to be more primitive of the two and may have features in common with the ancestral species of the whole *virilis* group [7,8,14]. Fortunately, the genome of *D. americana* is now completely annotated [27] and it is possible to perform BLAST searches for sequences of interest. We used this option to look for the presence of the 225 bp, 154 bp and 172 bp consensus sequences, which were previously detected in the *D. virilis* genome, in the annotated *D. americana* genomic sequences.

To our surprise, we failed to detect any sequences homologous to the *D. virilis* IGS 225 bp repeats in the *D. americana* genome. This family of repeats most likely appeared in and spread throughout the *D. virilis* genome after the separation of these species. It will be interesting to find out what repeated sequences are present within IGS of *D. americana* and other species of the group. It is of note that due to its repetitive nature the ribosomal gene region may be difficult to assemble, and this could be the reason why sequences homologous to the IGS 225 bp repeats have not been found so far in *D. americana*. Unless a scaffold is found with the whole intergenic region and without the repeat, this possibility cannot be altogether discarded. Interestingly, both sequenced genomes of *D. americana* do contain approximately the same number (approximately 150 copies) of 154 bp repeats, showing a high level of similarity (90-95%) with the consensus sequence of the 154 bp repeats from *D. virilis,* as described above. Because this sequence represents a fragment of the well-known *Helitron*-2 transposon, it is evident that multiple copies of this mobile element, possibly similarly truncated, are also present in the genome of *D. americana*. Therefore, invasion and massive amplification of *Helitron-2* apparently took place early in the evolution of the *virilis* phylad group. Similar situation was described in the species of *D. ananassae* subgroup where amplification of another family of 175–200 bp long repeats took place apparently exploring retroposition mechanism [24].

Similarly, our analysis enabled the detection of multiple copies of 172 bp repeats in the genome of *D. americana*. Thus, both investigated strains of *D. americana* contain approximately 180 copies belonging to this family of repeats. We can not say, however, whether the 172 bp repeats in *D. americana* are clustered, as is the case in the *D. virilis apterous* region, or scattered throughout the genome. We performed a BLAST search using the 172 bp consensus fragment as a query in other available sequenced *Drosophila* genomes and did not find any sequences with significant homology to the repeats. Thus, the tandem repeats are apparently specific for certain species of the *virilis* group of *Drosophila*. The comparison of the described medium-sized repeats between different species of the *virilis* group and other related species may be very helpful in understanding the function and origin of these repeated sequences and their possible role in the evolution of close species of *Drosophila*.

The described method has the potential to learn more about regions containing repeats. The knowledge about long repeats could be used to construct maps of these regions. Even though the digestion method would be laborious, it could potentially help to piece together a genomic sequence of the heterochromatic regions in particularly in species containing large proportion of repeats. Furthermore, the developed method may be used to detect the amplification of various transposable elements (“bursts”) by comparison of the restriction patterns of the individual strains and geographical populations of certain species with those of the basic sequenced species strain with partially or completely annotated genome.

## Conclusion

Using *in silico* digestion in combination with conventional restriction analysis and sequencing of repeated DNA fragments enabled us to isolate and characterize three highly abundant families of medium-sized repeats present in the *D. virilis* genome. These repeats comprise a significant portion of the *D. virilis* genome and may have important roles in genome function and structural integrity. Interestingly, two of the described families were also abundant in *D. americana*, which belongs to the same phylogenetic group. At the present time, we do not know whether these repeats were formed by unequal crossing-over events, replication slippage or the rolling-circle replication mechanism used in the propagation of *Helitron-*like transposons. This investigation emphasizes the validity and versatility of *in silico* digestion method for the detection and analysis of the multiple families of tandem repeats that often escape analysis in the process of genome assembling. Importantly, the suggested approach may help to shed light on the structure and composition of heterochromatic regions of the sequenced genomes and help to elucidate general trends in heterochromatin evolution.

## Methods

### Fly stocks

In our experiments, we used two strains of *D. virilis* and one *D. melanogaster* strain. *D. virilis* strain 160 is an old laboratory strain that carries recessive markers in all autosomes. A derivative of strain 160 was used to determine the genome sequence of *D. virilis*. The second *D. virilis* strain, strain 9, was used for comparison and represents the wild-type strain, caught in 1971 in Batumi, Georgia. In addition, we used the Oregon R strain of *D. melanogaster*. Flies of all species were reared on standard resin-sugar-yeast-agar medium containing proprionic acid and methylparaben as mold inhibitors.

### Isolation and analysis of genomic DNA and mRNA from *Drosophila* species

Genomic DNA was isolated from flies using a standard phenol-chloroform extraction technique. Hydrolysis reactions were performed for 2 hours, at optimal temperature, in 20 μl of the reaction mixture containing 2 μg of DNA, SE-buffers, as recommended by the manufacturer, and 1 μl of restriction enzyme. Gel electrophoresis using 8% agarose gel was conducted in Tris-acetate buffer to separate the DNA fragments. 2 μg of hydrolyzed DNA were loaded on agarose gel in each run. After electrophoresis, DNA bands were stained with ethidium bromide and photographed in UV light.

To determine 225 bp *Hpa*II fragments sequences, gel piece with visible 225 bp bands was excised out after electrophoresis. DNA fragments were isolated from gel pieces using QIAEX II Gel Extraction Kit (QIAGEN) and ligated with pUC19 plasmid linearized with *Sma*I. E.coli XL1-blue competent cells were transformed with obtained ligation mixture. Plasmid DNAs from the grown colonies were isolated using NucleoSpin Plasmid Kit (Macherey-Nagel). The sequences of insertions were determined using ABI Prism 310 Genetic Analyzer (Applied Biosystems).

Total RNA and poly (A)-RNA were extracted from the thoraxes or ovaries of adult flies, as previously described [28], and using the TRiZoL reagent (Sigma). The integrity of each RNA preparation was checked on ethidium bromide-stained 1.2% agarose/Mops-formaldehyde gels.

Radiolabeled probes (^32^P) were obtained by random priming of repeat-containing DNA fragments isolated from agarose gels. Five micrograms of poly (A) RNA were loaded in each lane of 1.2% agarose/Mops-formaldehyde gel. RNA transfer was performed on Hybond XL in 6xSSC overnight. The membranes were UV cross-linked. Hybridizations were performed overnight at 42°C in 50% formamide, using ultrasensitive hybridization buffer (Ambion).

### *In situ* hybridization with polytene chromosomes and cytological analysis

Salivary glands were dissected from *D. virilis* third-instar larvae in 45% acetic acid and squashed according to the described procedures [29]. For *in situ* hybridization studies, larvae were grown at 18°C, and a live yeast solution was added to the culture two days before the larvae were analyzed. The DNA probes described above were biotinylated by nick translation using biotin 14-dATP [29]. Chromosomal localizations were made using cytological photographic maps of *D.* virilis [30].

#### Sequence analysis and diagram plotting

The distribution diagrams of *Drosophila* genomic DNA digestion *in silico* at recognition sites of several restriction endonucleases were constructed according to the previously described techniques [1]. The Tandem Repeat Finder program [31] was used to obtain sequences of repeats that were 40–400 bp in length and arranged in tandem within the *Drosophila* genomic sequences [32].

## Competing interests

The authors declare that they have no competing interests.

## Authors’ contributions

MAC, DAG, VAC, VNT and JET carried out DNA hydrolysis, cloning and sequencing od DNA fragments. NGS and OGZ performed Northern analysis, ESZ performed in situ hybridization experiments. SKD and MBE coordinated the project and wrote the final manuscript. All authors have read and approved the final manuscript.

## Supplementary Material

Additional file 1: Figure S1 The alignment of 172 bp tandem repeats cluster located close to *apterous* gene of *D. virilis.* Deletions are indicated by yellow color, insertions are indicated by blue color, while grey color indicates hexanucleotide which does not have any homology with 172 bp consensus sequence. Click here for file
