# Supplementary material for: Medium-sized tandem repeats represent an abundant component of the Drosophila virilis genome
Source: BMC Genomics. 2013 Nov 9;14:771. doi: 10.1186/1471-2164-14-771 (PMC3833285; doi:10.1186/1471-2164-14-771)
Supplement: Additional file 1: Figure S1 — The alignment of 172 bp tandem repeats cluster located close to apterous gene of D. virilis. Deletions are indicated by yellow color, insertions are indicated by blue color, while grey color indicates hexanucleotide which does not have any homology with 172 bp consensus sequence. [file 1471-2164-14-771-S1.doc]

TACCCGCTATTATTC-TATATAGACATAGATCGAAAACTCCCAACCCTATAACTCGACCAAAACTCAACCGATTTTCATAAGGTTTAATTTTTTGTTCATGGTT---TGACCTCTATATCAATCTGGCATATAAATCTGAAAACTTTATTTTTGGTCAAAATTCATGTGAGAACGG

TACCATGAAATATCC-TACATAGACATAGGTCGAAAATTCCCAACCCCATAACTTGGCCAAAACTCAACCGATTTTCATAAGGTATACATTTTTGTTCATGGTT---TGACCTCTATATCAATCTGGCATATAAATCTGACAACTTTATTTTTGGTCAAAATTCATGTGAAAATGG

TACCATCAAATATCC-TACATAGACATAGGTCGAAAATTCCCAACCCCATAACTTGGCCAAAACTCAACCGATTTTCATAAGGTTTAACTTTTTGTTCATGGTT---TGACCTCTATATCAATCTGGCATATAAATCTGAAAACTTCATTTTTGGTCAAAATTCATGTGAGAACGG

TACCATGAAATATCC-TACATAGACATAGGTCGAAAATTCCCAACCCCATAACTTGGCCAAAACTCAACCGATTTTCATAAGGTTTAACTTTTTGTTCATGGTT---TGACCTCTTTATCAATCTGGCATATAAATCT--AAACTTTATTTTTGGTCAAAATTCGTGTGAGAACGG

TACCATGAAATATCC-TACATAGACATAGGTCGAAAATTCCCAACCCCATAACTTGGCCAAAACTCAACCGATTTTCATAAGGTTTAACTTTTTGTTCATGGTT---TGACCACTATATCAATCTGGCATATAAATCTGACAACATTATTTTTGGTTAAAATTCATGTGAAAATGG

TACCATCAAATATCC-TACATAGACATAGGTCGAAAATTCCCAACCCCATAACTCGGCCAAAACTCAACCGATTTTCATAAGGTATACATTTTTGTTCATGGTT---TGACCTCAATATCAATCTGGCATATTAATCTGACAACTTTATTTTTGGTCAAAATTCATGTGTAAATGT

TACCACGAAATATCC-TACATATTTATCAA

-----------ATCC-TACACAGACATAGGTCGAAAATTCCCAACCCCATAACTTGGCCAAAACTCAACCGATTTTCATAAGGTTTAACTTTTTGTTCATGGTT---TGACCACTATATCAATCTGGCATATAAATCTGACAACTTTATTTTTGGTCAAAATTCATGTGAAAATGG

TACCATCAAATATCC-TACATAGACATAGGTCGAAAATTCCCAACCCCATAACTTGGCCAAAACTCAACCGATTTTCATAAGGTTTAACTTTTTGTTCATGGTT---TGACCTCTATATCAATCTGGCATATAAATCTGACAACTTTATTTTTGGTCAAAATTCATGTGAAAGTGG

TACCATCAAATATCC-TACATAGACATAGGTCCAACATTCCCAACCCCATAACTCGGCCAAAACTCAACCGATTTTCATAAGGTATACATTTTTGTTCATGGTT---TGACCTCAATATCAATCTGGCATATAAATCTGACAACTTTATTTTTGGTCAAAATTCATGTGTAAATGT

TACCATGAAATACACATACACAGACATAGGTCGAACATTCCCAACCCCATAACTCGGCCAAAACTCAACCGATTTTCATAAGGTTTAACTTTTTGTTCATGGTT---TGACCTCAATATCAATCTGGCATATAAATCTGACAACTTTATTTTTGGTCAAAATTCATGTGAAAATGG

TACCATGAAATATCC-TACATAGACATAGGTCGAATATTCCCAACCCTATAACTCGGCCAAAACTCAACCGATTTTCATAAGGAATACATTTTTGTTCATGGTT---TGACCTCTATATCGATTTGGCATATAAATCTGACAACTTTATTTTTGGTCAAAATTCATGTGAAAATGG

TACCATCAGATATCC-TACTTTTTATCAA

-----------ATCC-TACACAGACATAGGTCGAACATTCCCAACCCCATAACTCGGCCAAAACTCAACCGATTTTCATAAGGTTTAACTTTTTGTTCATGGTT---TGACCTCAATATCAATCTGGCATATAAATCTGACAACTTTATTTTTGGTCAAAATTCATGTGAAAATGG

TACCATGAAATATCC-TACATAGACATAGGTCGGAAATTCCCAACCCTATAACTCGGCCAAAACTCAACCGATTTTCATAAGGAATACGTTTTTGTTCATGGTT---TGACCTCTATATCGATTTGGCATATAAATTTGACAACTTTATTTTTGGTCAAAATTCATGTGAAAATGG

TACCATGAAATATCC-TACATAGACATAGGTCGAACATTCCCAAGCCCATAACTCGGCCAAAACTCAACCGATTTTCATAAGGTTTAACTTTTTGTTCATGGTT---TGACCTCAATATCAATCTGGCATATAAATCTGACAACTTTATTTTTGGTCAAAATTCATGTGTATATGT

TACCATCAAATATCC-TACATAGACATTGGTCGAAAATTCCCAACCCCATAACTTGGCCAAAACTCAACCGATTTTCATAAGGTTTAACTTTTTGTTCATGGTT---TGACCTCTATATCAATCTGGCATATAAATCTGACAACTTTATTTTTGGTCAAAATTCATGTGAAAATGG

TACCATGAAATATCC-TACATAGACATAGGTCGAATATTCCCAACCCTATAACTCGGCCAAAACTCAACCGATTTTCATAAGGAATACATTTTTGTTCATGGTT---TGACCTCTATATCGATTTGGCATATAAATCTGACAACTTTATTTTTGGTCAAAATTCATGTGAAAATGG

TACCATCAGATATCC-TACTTTTTATCAA

-----------ATCC-TACATAGACATAGGTCGAATATTCCCAACCCTATAACTCGGCCAAAACTCAACCGATTTTCATAAGGAATACATTTTTGTTCATGGTT---TGACCTCTATATCGATTTGGCATATAAATCTGACAACTTTATTTTTGGGCAAAATTCATGTGAAAATGG

TATCATCAGATATCC-TACTTTTTATCAA

-----------ATCC-TACACAGACATAGGTCGAACATTCCCAACCCCATAACTCGGCCAAAACTCAACCGATTTTCATAAGGTTTAACTTTTTGTTCATGGTT---TGACCTCAATATCAATCTGGCATATAAATCTGACAACTTTATTTTTGGTCAAAATTCATGTGAAAATGG

TACCATGAAATATCC-TACATAGACATAGGTCGGAAATTCCCAACCCTATAACTCGGCCAAAACTCAACCGATTTTCATAAGGAATACGTTTTTGTTCATGGTT---TGACCTCTATATCGATTTGGCATATAAATTTGACAACTTTATTTTTGGTCAAAATTCATGTGAAAATGG

TACCATGAAATATCC-TACATAGACATAGGTCGAAAATTCCCAACCCCATAACTTGGCCAAAACTCAACCGATTTTCATAAGGTTTAACTTTTTGTTCATGGTT---TGACCTCTATATCAATCTGGCATATAAATCTGACAACTTTATTTTTGGTCAAAATTCATGTGAAAATGG

TACCATCAAATATCC-TACATAGACATTGGTCGAAAATTCCCAACCCCATAACTTGGCCAAAACTCAACCGATTTTCATAAGGTTTAACTTTTTGTTCATGGTT---TGACCTCTATATCAATCTGGCATATAAATCTGACAACTTTATTTTTGGTCAAAATTCATGTGAAAATGG

TACCATGAAATATCC-TACATAGACATAGGTCGAATATTCCCAACCCTATAACTCGGCCAAAACTCAACCGATTTTCATAAGGAATACATTTTTGTTCATGGTT---TGACCTCTATATCGATTTGGCATATAAATCTGACAACTTTATTTTTGGTCAAAATTCATGTGAAAATGG

TACCATCAGATATCC-TACTTTTTATCAA

-----------ATCC-TACACAGACATAGGTCGAACATTCCCAACCCCATAACTCGGCCAAAACTCAACCGATTTTCATAAGGTTTAACTTTTTGTTCATGGTT---TGACCTCAATATCAATCTGGCATATAAATCTGACAACTTTATTTTTGGTCAAAATTCATGTGAAAATGG

TACCATGAAATATCC-TACATAGACATAGGTCGGAAATTCCCAACCCTATAACTCGGCCAAAACTCAACCGATTTTCATAAGGAATACGTTTTTGTTCATGGTT---TGACCTCTATATCGATTTGGCATATAAATTTGACAACTTTATTTTTGGTCAAAATTCATGTGAAAATGG

TACCATGAAATATCC-TACATAGACATAGGTCGAACATTCCCAAGCCCATAACTCGGCCAAAACTCAACCGATTTTCATAAGGTTTAACTTTTTGTTCATGGTT---TGACCTCAATATCAATCTGGCATATAAATCTGACAACTTTATTTTTGGTCAAAATTCATGTGTATATGT

TACCATCAAATATCC-TACATATTTATCAA

-----------ATCC-TACACAGGCATAGGTCGAACATTCCCAACCCCATAACTCGGCCAAAACTCAACCGATTTTCATAAGGTTTAACTTTTTGTTCATGGTT---TGACCTCTATATCAATCTGGCATATAAATCTGACAACTTTATTTTTGGTCAAAATTCATGTGAAAATGG

TACCATCAAATATCC-TACATAGACATAGGTCCAACATTCCCAACCCCATAACTCGGCCAAAACTCAACCGATTTTCATAAGGTATACATTTTTGTTCATGGTT---TGACCTCAATATCAATCTGGCATATTAATGTGACAACTTTATTTTTGGTCAAAATTCATGTGTAAGTGT

TACCATGAAATATCC-TACATATTTATCAA

-----------ATCC-TACACAGACATAGGTCGAACATTCCCAACCCCATAACTCGGCCAAAACTCAATCGATTTTCATAAGGTTTAACTTTTTGTTCATGGTT---TGACCTCAATATCAATCTGGCATATAAATCTGACAACTTTATTTTTGGTCAAAATTCATGTGAAAATGG

TACCATGAAATATCC-TACATAGACATAGGTCGAAAATTCCCAACCCCATAACTCGGCCAAAACTCAGCCGATTTTTATAAGGTATAGCTTTTTGTTCATGGTT---TGACCACAATATCAATCTGGCATATAAATCTGACAACTTTATTTTTGGTCAAAATTCATGTGAAAATGG

TACCATGAAATATCC-TACATAGACATAGGTCGGAAATTCCCAACCCTATAACTCGGCCAAAACTCAACCGATTTTCATAAGAAATACATTTTTGTTCATGGTT---TGACCTCTATATCGATTTGGCATATAAATCTGACTACTTTATTTTTGGTCAAAATTCATGTGAAAATGG

TACCATCAGATATCC-TACTTTTTATCAA

-----------ATCC-TACACAGACATAGGTCGAAAATTCCCAACCCCATAACTCGGCCAAAACTCAACCGATTTTCATAAGGTTTAACTTTTTGTTCATGGTT---TGACCTCAATATCAATCTGGCATATAAATCTGACAACTTTATTTTTGGTCAAAATTCATGTGAAAATGG

TACCATGAAATATCC-TACATAGACATAGGTCGGAAATTCCCAACCCTATAACTCGGCCAAAACTCAACCGATTTTCATAAGGAATACATTTTTGTTCATGGTTGTTTGACCTCTATATCGATTTGGCATATAAATCTGACAACTTTATTTTTGGTCAAAATTCATGTGAAAATGG

TACCATCATATATCC-TACATAGACATAGGTCGAACATTCCCAAGCCCATAACTCGGCCAAAACTCAACCGATTTTCATAAGGTTTAACTTTTTGTTCATGGTT---TGACCTCAATATCAATCTGGCATATAAATCTGACAACTTTATTTTTGGTCAAAATTCATGTGTATATGT

TACCATCAAATATCC-TACATATTTATCAA

-----------ATCC-TACACAGACATAGGTCGAACATTCCCAACCCCATAACTCGGCCAAAACTCAACCGATTTTCATAAGGTTTAACTTTTTGTTCATGGTT---TGACCTCTATATCAATCTGGCATATAAATCTGACAACTTTATTTTTGGTCAAAATTCATGTGAAAATGG

TACCATGAAATATCG-TACATAGACATAGGTCGGAAATTCCCAACCCTATAACTCGGCCAAAACTCAACCGATTTTCATAAGGAATACATTTTTGTTCATGGTT---TGACCTCTATATCGATTTGGCATATAAATCTGACAACTTTATTTTTGGTCAAAATTCATGTGAAAATGG

TACCATCATATATCC-TACATAGACATAGGTCGAACATTCCCAAGCCCATAACTCGGCCAAAACTCAACCGATTTTCATAAGGTTTAACTTTTTGTTCATGGTT---TGACCTCAATATCAATCTGGCATATAAATCTGACAACTTTATTTTTGGTCAAAATTCATGTGTATATGT

TACCATCAAATATCC-TACATATTTATCAA

-----------ATCC-TACACAGACATAGGTCGAACATTCCCAACCCCATAACTCGGCCAAAACTCAACCGATTTTCATGAGGTTTAACTTTTTGTTCATGGTT---TGACCACAATATCAATCTGGCATATAAATCTGACAACTTTATTTTTGGTCAAAATTCATGTGAAAATGG

TACCATGAAATATCG-TACATAGACATAGGTCGGAAATTCCCAACCCTATAACTCGGCCAAAACTCAACCGATTTTCATAAGGAATACATTTTTGTTCATGGTT---TGACCTCTATATCGATTTGGCATATAAATCTGACTACTTTATTTTTGGTCAAAATTCATGTGAAAATGG

TACCATGAAATATCC-TACATAGACATAGGTCGACAATTCCCAACCCCATAACTCAGACAAAACTCAACCGATTTTCATAAGATTTAACTTTTTGTTCATGGTT---TGACCTCAATATCAATCTGGCATATAAATCTGACAACTTTATTTTTGGTCAAAATTCATGTGTATATGT

TACCATCAAATATCC-TACATATTTATCAA

-----------ATCC-TACACAGACATAGGTCGAACATTCCCAACCCCATAACTCGGCCAAAACTCAAACGATTTTCATAAGGTTTAACTTTTTGTTCATGGTT---TGACCGCAATATCAATCTGGCATATAAATCTGACAACTTTATTTTTGGTCAAAATTCATGTGAAAATGG

TACCATGAAATATCC-TACATAGACATAGGTCGGAAATTCCCAACCCTATAACTCGGCCAAAACTCAACCGATTTTCATAAGGAATACAATTTTGTTCATGGTT---TGACCTCTATATCGATTTGGCATATAAATCGGACAACTTTATTTTTGGTCAAAATTCATGTGAAAATGG

TACCATGAAATATCC-TACATAGACATAGGTCGACAATTCCCAACCCCATAACTCAGACAAAACTCAACCGATTTTCATAAGATTTAACTTTTTGTTCATGGTT---TGACCTCAATATCAATCTGGCATATAAATCTGACAACTTTATTTTTGGTCAAAATTCATGTGTATATGT

TACCATCAAATATCC-TACATATTTATCAA

-----------ATCC-TACACAGGCATAGGTCGAACATTCCCAACCCCATAACTCGGCCAAAACTCAACCGATTTTCATAAGGTTTAACTTTTTGTTCATGGTT---TGACCTCTATATCAATCTGGCATATAAATCTGACAACTTTATTTTTGGTCAAAATTCATGTGAAAATGG

TACCATGAAATATCC-TACATAGACATAGGTCGAAAATTCCCAAGCCCATAACTCGGCCAAAACTCAACCGATTTTCATAAGGTATACATTTTTGTTCATGGTT---TGACCTCAATATCAATCTGGCATATAAATCTGACAACTTTATTTTTGATCAAAATTAATGTGAAAATGA

TACCATGAGATATCC-TACATA

**Additional file 1: FigureS1. The alignment of 172 bp tandem repeats cluster located close to *apterous* gene of *D. virilis***

Deletions are indicated by yellow color, insertions are indicated by blue color, while grey color indicates hexanucleotide which does not have any homology with 172 bp consensus sequence.
